# Nursing home staff experiences of implementing mentorship programmes: A systematic review and qualitative meta‐synthesis

**DOI:** 10.1111/jonm.12876

**Published:** 2020-02-03

**Authors:** Lulu Liao, Lily Dongxia Xiao, Huijing Chen, Xin Yin Wu, Yinan Zhao, Mingyue Hu, Hengyu Hu, Hui Li, Xiufen Yang, Hui Feng

**Affiliations:** ^1^ Xiangya School of Nursing Central South University Changsha China; ^2^ College of Nursing and Health Sciences Flinders University Adelaide SA Australia; ^3^ Department of Epidemiology and Biostatistics Xiangya School of Public health Central South University Changsha China; ^4^ Third Xiangya Hospital of Central South University Changsha China; ^5^ Xiangya‐Oceanwide Health Management Research Institute Central South University Changsha China

**Keywords:** mentorship, nursing home, qualitative meta‐synthesis, systematic review

## Abstract

**Aim:**

To determine nursing home staff experiences in mentorship programmes, and staff perceptions of the enablers and barriers to implement mentorship programmes.

**Background:**

Mentorship programmes are perceived as playing an important role in improving the quality of care in nursing homes. However, little is known about research evidence across the global about staff's experiences in the programmes.

**Methods:**

A search for studies published from the earliest available date to April 2019 was undertaken. Two reviewers performed data extraction and an appraisal of eight studies using tools from the Joanna Briggs Institute. A pragmatic meta‐aggregative approach was applied to synthesise the findings. The qualitative research that was included was analysed to identify 63 findings that were organised into 12 categories and combined into three syntheses.

**Results:**

The implementation of effective mentorship programmes is influenced by three factors: mentor capability, opportunity in the mentorship programmes, and motivation in the mentorship programmes.

**Conclusions:**

There are a number of studies of nursing home staff experiences of mentorship programmes. However, systematic reviews that synthesise findings in this field are lacking. It is crucial to tailor the programme design to suit each unique nursing home care setting. We identified barriers and enablers, and learned that no barriers are insurmountable.

**Implications for Nursing Management:**

Findings will inform nurse managers of an ideal environment for the implementation of a successful mentorship programme. Nursing homes need to establish and sustain mentorship programmes to help improve workforce capacity in delivering high‐quality care for residents.

## 1 INTRODUCTION

The number of older adults who lose the ability to care for themselves continues to increase with advancing age (Пaин, 2017). Home‐based care, which mainly consists of personal care, cannot adapt to the current needs of older adults, and responsibility for the care of older adults is gradually transferred to society as a whole (Wiederhold, Riva, & Graffigna, 2013). At the same time, in the face of huge demands in societies with ageing populations, staffing shortages and a high turnover of nursing home staff have become major challenges (Kendall‐Raynor, 2016; Vermeerbergen, Van Hootegem, & Benders, 2017). In addition, insufficient trainers and a lack of standardized training are important factors affecting the quality of care (Woo, Milworm, & Dowding, 2017). There are an increasing number of studies on residents’ experiences and satisfaction with nursing home care (Vaismoradi, Wang, Turunen, & Bondas, 2016). Hence, more emphasis should be placed on nursing staff education, training and supervision in an attempt to promote quality of care.

A mentorship programme is defined as a staff development programme that adopts a series of organized methods to train and educate employees using mentors who are experienced nursing staff. Mentors serve as a resource and guide to encourage staff members to develop themselves personally or professionally in an area of importance to them, and to help them acquire confidence in their work (Anderson & Shannon, 1988). Mentorship projects are symbiotic and reciprocal, providing a win–win situation for both mentor and mentee, and have demonstrated success in improving the quality of care in nursing homes (Feng et al., 2018; Morrow, 2009). Numerous studies have shown that mentorship programmes can promote nurse retention, improve nursing staff ability and professionalism and establish a supportive learning environment, resulting in positive resident care outcomes (Burr, Stichler, & Poeltler, 2011; Schoonbeek & Henderson, 2011; Schroyer, Zellers, & Abraham, 2016; Ward & McComb, 2018). Although there are differences between mentors, preceptors and champions, all serve as helpers who assist mentees or preceptees in improving their knowledge and skills (Agrell‐Kann, 2015; Greggs‐McQuilkin, 2004; Wensel, 2006; Woo et al., 2017). Therefore, this systematic review focuses on all people who play a similar role.

To date, there have been a considerable number of papers on mentorship projects in hospital, but we found relatively few studies conducted in a nursing home care setting, indicating a research disparity in this important field of study between acute care hospitals and nursing homes (Aaron, 2011; Omansky, 2010; Zhang, Qian, Wu, Wen, & Zhang, 2016). Compared with the resources typically available in hospitals, nursing homes are usually perceived as resource‐poor settings for staff development. In nursing homes, registered nurses are in the minority and the majority of staff are unlicensed nursing assistants who have had limited vocational training prior to employment (Woo et al., 2017). These staff profiles generate challenges for nurse managers in identifying suitable mentors for staff. Furthermore, the nursing home learning environment is perceived as poor in supporting mentoring activities due to staff shortages, heavy workload, inadequate routines and leadership styles that do not acknowledge staff as a key resource (Husebo, Storm, Vaga, Rosenberg, & Akerjordet, 2018; Skaalvik, Normann, & Henriksen, 2011). In addition, the quality of care in nursing homes is lower than in hospitals, with low hygiene standards and inadequate nursing care documentation (Husebo et al., 2018). Mentees perceive that these factors prevent them from participating in learning activities (Brynildsen, Bjork, Berntsen, & Hestetun, 2014).

Improving nursing home care quality while reducing costs is a topic that has attracted increased attention in clinical mentorship studies in nursing homes (Feng et al., 2018). Qualitative research is a desirable research design to gain insight into the experiences of nursing home staff. This type of research plays a vital role in informing the meaningfulness, feasibility and acceptability of education and training interventions for nursing home staff (Sandelowski & Barroso, 2003). While studies on implementing education and training programmes for nursing home staff, including mentorship activities, have been abundant, systematic reviews that synthesize the findings in this field are lacking (Chen & Lou, 2014; Edward, Ousey, Playle, & Giandinoto, 2017; Woo et al., 2017). This systematic review addresses a gap in the literature by synthesizing qualitative studies to gain a new understanding of nursing home staff experiences, and their perceptions of implementing mentorship programmes, from a broader range of studies from around the world.

### 1.1 AIM

In this review, we aimed to determine nursing home staff experiences in mentorship programmes, and their perceptions of enablers and barriers when implementing mentorship programmes.

## 2 METHOD

### 2.1 Research design

The systematic review of qualitative research was based on the meta‐synthesis approach of the Joanna Briggs Institute (JBI) (Hannes & Lockwood, 2011). Findings from the studies that were included were categorized based on similarity of meaning. These categories were subjected to a meta‐synthesis to produce a series of synthesis findings that can be used as a basis for evidence‐based practice (Jordan, Lockwood, Munn, & Aromataris, 2018). This method aims to produce a new, integrative interpretation of qualitative research findings, which is more substantive and meaningful than that of individual investigations (Finfgeld, 2003). The systematic review protocol is registered with PROSPERO: CRD42019131514.

### 2.2 Search strategy

We searched the following six databases from the earliest available date to April 2019: CINAHL, Ovid MEDLINE(R), Ovid Embase, Scopus, Web of Science and PsycINFO. A three‐step search strategy was used in this paper. An initial limited search of MEDLINE and CINAHL was undertaken, followed by an analysis of the text contained in each title and abstract, and of index terms used to describe the article. A second extensive search, using all identified key words and index terms, was then undertaken. Lastly, the references list of all identified reports and articles was searched for additional studies. The research was not limited to the English language. The initial key search terms used were as follows: mentor; mentorship; mentee; champion; preceptor; nursing home; residential aged care facilities; homes for the aged. The full search strategy is provided in Appendix S1.

### 2.3 Eligibility criteria and study selection

The inclusion criteria of this systematic review are as follows: (a) primary studies using qualitative methodology or mixed methods; (b) the context was nursing homes, including residential aged care facilities, long‐term care, health care facilities. Nursing schools or hospitals were excluded as a setting; (c) the focus was on staff experiences with being set‐up with mentors/preceptors/champions. We excluded literature that only used quantitative methods to investigate similar phenomena.

**Figure 1:**
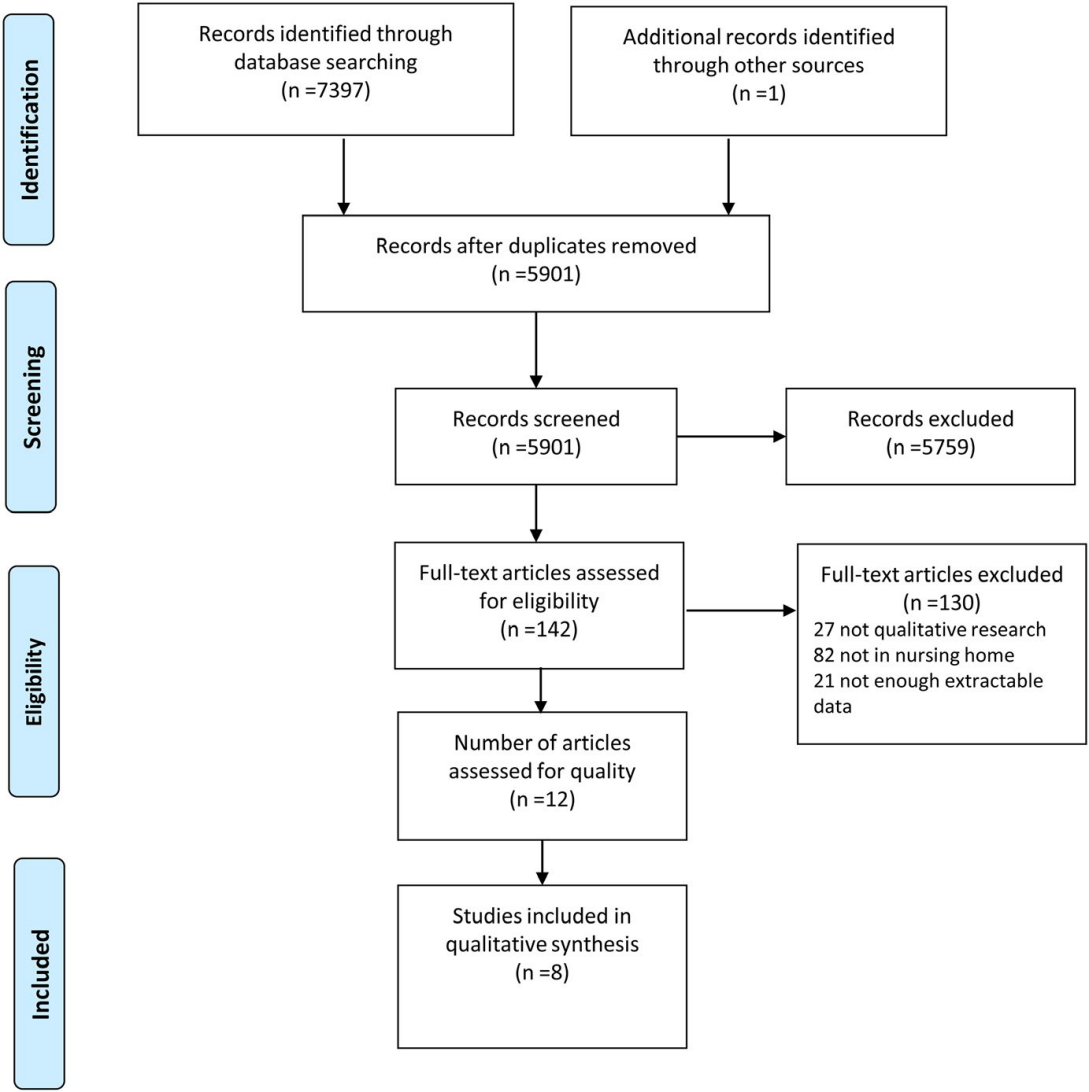
Flowchart of the search process (PRISMA) [Colour figure can be viewed at http://wileyonlinelibrary.com]

The initial searches located 7,397 publications that were imported into Endnote X9 software. There were 1,496 duplicates identified through the Endnote function and hand searching in Endnote, leaving 5,901 papers that were assessed by title and abstract relevance. The screening resulted in 142 articles meeting the selection criteria, and the full text of these articles was retrieved for further assessment. After reading the full text, 12 papers were eligible for quality appraisal. Figure 1 shows the search strategy. The screening process was undertaken by two reviewers (LLL and CHJ), and there were no disagreements.

### 2.4 Quality assessment

The critical appraisal instruments from the Joanna Briggs Institute Qualitative Assessment and Review Instrument (JBI‐QARI) were used to appraise each paper's research methodology rigour (Hannes, Lockwood, & Pearson, 2010). A cut‐off point of six out of the 10 questions answered as “yes” was established to ensure that lower‐quality studies were excluded (Aromataris & Pearson, 2014; Tacconelli, 2010). During the quality assessment process, four studies were excluded due to a lack of detail for the assessment of eligibility (Appendix S2). Any disagreements between the two reviewers (LLL and CHJ) were resolved through discussion, or with a third reviewer on the team. Table 1 provides a quality assessment of the papers that were included in the review.

**Table 1:** Quality assessment of included studies

Citation	Q1	Q2	Q3	Q4	Q5	Q6	Q7	Q8	Q9	Q10	Score
Kaasalainen S et al. (2015)	U	Y	Y	N	Y	N	Y	N	Y	Y	6
Kaasalainen S et al. (2016)	U	Y	Y	Y	Y	N	N	Y	Y	Y	7
Ploeg et al. (2010)	U	Y	Y	Y	Y	N	N	Y	Y	Y	7
Aubry et al. (2012)	U	Y	Y	Y	Y	N	N	Y	Y	Y	7
Cadmus et al. (2016)	U	Y	Y	Y	Y	N	N	N	Y	Y	6
Rohatinsky and Jahner (2016)	Y	Y	Y	N	Y	N	N	Y	Y	Y	7
Ryan and McAllister (2017)	U	Y	Y	Y	Y	N	N	Y	Y	Y	7
DeCicco (2008)	U	Y	Y	Y	Y	N	N	Y	N	Y	6

### 2.5 Data extraction and synthesis

Data were extracted from papers included in this review using a standardized data extraction tool from JBI‐QARI. The first author extracted data from the eight studies. The reviewers carefully read the papers that were included and extracted the relevant findings from the primary studies. Two reviewers independently appraised each finding and attributed a level of credibility to each one. The levels of credibility were as follows: unequivocal (U)—relates to findings beyond a reasonable doubt; credible (C)—relates to findings that are interpretations, plausible in light of the data and theoretical framework; and unsupported (Un)—when the findings are unsupported by the data. There were no disagreements as to the credibility levels. We aggregated the findings into different categories based on similarity of meanings; then, the categories were subjected to a meta‐synthesis to generate synthesized findings by meta‐aggregation (Lockwood, Munn, & Porritt, 2015). The first author led the meta‐synthesis process. The process was repeated when reviewing findings/categories/synthesized findings. If in doubt, we consulted the original literature and held a group discussion to reach an agreement. We used the ConQual tool to evaluate confidence in the synthesized findings (Munn, Porritt, Lockwood, Aromataris, & Pearson, 2014).

## 3 FINDINGS

### 3.1 Characteristics of the studies

A total of eight research studies were included in the review. Of this total, five were qualitative studies and three were mixed method studies with an analysis of qualitative results. All papers that were included were published between 2010 and 2018. Among the eight papers, six were from Canada, and two were from the United States and Australia, respectively. Detailed results are presented in Appendix S3.

### 3.2 Meta‐synthesis of qualitative data

A total of 63 findings were extracted from the eight included papers (Appendix S4): 44 unequivocal and 19 credible. These findings were aggregated into 12 categories based on the similarity of meanings; then, these categories were meta‐aggregated into three synthesized findings (Table 2). The results of the meta‐synthesis are shown in Appendix S5.

**Table 2:** Themes of Meta‐synthesis

Synthesized findings	Category	Kaasalainen S et al, 2015	Kaasalainen, S et al, 2016	Ploeg et al, 2010	Aubry et al, 2012	Cadmus et al, 2016	Rohatinsky and Jahner, 2016	Ryan and McAllister, 2017	DeCicco, 2008
Mentor capability	Selecting mentors	√	√			√	√		√
	The role of mentor	√	√	√	√		√		√
	Training and education			√				√	
Opportunities in the mentorship programme	Mentor matching						√	√	√
	Trusting relationships	√					√		
	Diverse mentoring styles	√	√	√	√				
	The lack of defined accountability						√		√
	Time constraints				√	√		√	√
	Unavailable mentors						√		√
Motivation in the mentorship programme	Proactivity			√		√	√	√	√
	Support and reward of management	√					√		√
	Traditional hierarchy							√	

#### 3.2.1 Synthesized finding 1: Mentor capability

It is important to recognize that mentor capability has a deep impact on the development of mentorship education programmes as well as on participant experiences. Selecting suitable mentors, defining the mentor's role and improving mentor capability through standardized training and education are crucial factors in establishing and maintaining successful mentorship programmes.

Mentors should be approachable, dependable and knowledgeable, with strong communication skills and clinical expertise. It is also recommended that suitable mentors should have a good work ethic, and not simply be the bossy type who wants to control everything.

I think that [NPs] are able to display a higher level of knowledge and understanding and so they gain the respect of the nurses who see them that way and not necessarily just another pair of hands… (Kaasalainen et al., 2015, p. 85)

the staff highly regarded the personal attributes of the NPs, which included being approachable, dependable, knowledgeable and having clinical expertise… (Kaasalainen et al., 2016, p. 162)

Mentors play many roles and serve as educators, helping new staff adapt to their workplace roles as well as meeting their learning needs.

Mentorship to me is when a seasoned nurse takes a person just entering their career under their wing and just tries to help them with the growing process. (Rohatinsky & Jahner, 2016, p. 4)

Meanwhile, mentors act as clinical leaders who facilitate new staff transitioning into the workplace environment and lead interdisciplinary teams. Mentors also serve as a communication liaison between staff and the experts.

Champions indicated that although their team work involved mostly nurses, they also interacted with a host of other interdisciplinary team members. “anything relating to OT (Occupational Therapy) or Physio (Physiotherapy), our members of the team would then take that back to those people and discuss things that were going on… We spoke with [nurses] on the floor”. (Ploeg et al., 2010, p. 246)

Mentors also expressed a need for training and education to improve their capability. Unmet learning needs indicate a lack of support for mentors to implement the programme.

…I need to learn more about giving and receiving feedback. (Louise)

… I need more training in reflective practice. (Helen)

…If I could assess students properly that would help me give better feedback. (Donna) (Ryan & McAllister, 2017, p. 4)

#### 3.2.2 Synthesized finding 2: Opportunity in the mentorship programmes

It is crucial to identify factors affecting staff's opportunities to participate in the mentorship programmes in the working environment. The impact of favourable opportunities can be related to suitable mentor matching, trusting relationships and diverse styles of mentoring. The lack of opportunity to learn in mentorship programmes can be connected to time constraints, unavailable mentors and a lack of defined accountability for mentors.

Suitable mentor matching plays an important role in promoting mentee engagement. When matching mentors with mentees, mentors should not be assigned to mentees through a managerial approach, but mutually identified by both parties.

Preceptees identified that they were not always paired with one consistent preceptor. One new hire stated “I wasn't really paired with anyone in particular. I think I had three preceptors.” (DeCicco, 2008, p. 21)

It is suggested that mentees use personality questionnaires to assist in more appropriate matching.

I knew their personality, I got to know them a bit better. And I think with rural [nursing] you're going to be working with the same seven RNs all the time. It makes a big difference knowing who you get along with. If I was assigned to somebody who I didn't feel comfortable with, it would have made a complete difference. (Rohatinsky & Jahner, 2016, p. 8)

Mentees are also encouraged to actively express their ideas in order to match mentees from different practice domains.

Participants felt protégés should have input with respect to mentor selection. One newer nurse suggested getting to know staff first before establishing a formal mentorship with an individual… (Rohatinsky & Jahner, 2016, p. 8)

Establishing a trusting and positive relationship between mentors and mentees is conducive to implementing a successful mentorship programme. Trust needs to be established before a relationship can be fostered, and mentors and mentees should get to know one another first. If anyone in such a relationship does not trust the other person, it can become an obstacle to successful mentoring. It was perceived that a personality connection between the mentor and mentee also played an important role.

And they were just so just friendly and embracing of [me]. They just made me feel really appreciated that I was coming to work casual. One of the staff members gave me a hug my first day and she was just so happy that I was here. I think that just that positive environment makes you want to stay. And so that kind of begins the relationship off in a good way. (Rohatinsky & Jahner, 2016, p. 6)

Despite practical limitations, mentors can create a positive learning environment for mentees in diverse styles of mentoring. In addition to regular training, meeting with clinical leaders on a daily basis, transmission of informal work strategies and using educational poster boards or an electronic system were other good ways to promote staff learning. On the other hand, limited communication between employees and conflicts between team members were not conducive to learning.

I think putting those [pain assessment tools] onto a computerized version was just a lot easier for the staff too as a reminder to automatically do that. And it just made them think of it, too. It just made a lot of people more aware of pain and what it looked like. (Kaasalainen et al., 2015, p. 83)

I tell those who come here for meal times to feed two at a time…I once served three at once. You put two on either side of you and feed them alternately to avoid losing any time.” Such strategies, created and used by nursing assistants and transmitted from experienced nursing assistants to new recruits, illustrate the incredible resourcefulness of these nursing assistants as they tried to meet their workload requirements. (Aubry et al., 2012)

In some cases, institutions lack clearly defined accountability for individuals, including new employees and mentors. This may result in adverse impacts on mentoring and disturb the normal work of mentors.

The workflow analysis revealed that one of the main challenges with our preceptorship program was a lack of clearly defined accountability. Participants suggested that the new model must clearly identify singular accountability within each service delivery center (SDC) for the new hire and their preceptorship experience. (DeCicco, 2008, p. 20–21)

Many mentors and mentees emphasized that time constraints exerted adverse effects on effective mentorship. Mentors indicated that they did not have enough time to make themselves available to guide mentees. Limited time also results in work behaviours that do not coincide with staff learning during training.

Time is the issue here. I don't have time to show/teach students properly or to do courses. We get no recognition and no paid study leave. (Ryan & McAllister, 2017, p. 5)

during our training, we are told to be careful, to take whatever time we need…that is why it is hard when we start working in the organization.… (Aubry et al., 2012)

In nursing homes, there is typically a large number of mentees who need to be guided, but the availability of mentors is limited. As a majority of nursing home staff are not licensed health care professionals, the demand for professional mentors often exceeds the supply. What is more, due to a lack of available mentors, some facilities choose people who are not interested in this position or have not been through a training process to become a mentor.

It was also discovered that nurses who had not been through the training process, and who were not particularly interested in being preceptors, were still asked to take on the role due to a lack of trained preceptors within the SDC. (DeCicco, 2008, p. 21)

They also recognized several factors that were impeding efficacy of the Pain Team and pain management including: the large number of staff at the home in need of pain education, the lines of communication among staff, and the limited NP availability in the LTC home. (Kaasalainen et al., 2016, p. 164)

#### 3.2.3 Synthesized finding 3: Motivation in the mentorship programmes

Motivation is an individual's automatic and reflexive mechanisms that trigger or inhibit behavioural changes in mentorship programmes. After the implementation of a mentorship programme, the motivation of staff behavioural changes is mainly influenced by proactivity, management support, rewards and hierarchy.

Proactivity is reflected in the fact that most mentors were willing to share their knowledge, and they felt very proud when a new employee gained more professional skills.

The preceptors noted they had an increase in personal pride in being able to share knowledge. They also identified they had been exposed to new methods of learning such as the simulation exercises. (Cadmus et al., 2016, p. 238)

The nurse residents and new graduate nurses are also supportive of the programme.

The nurse residents were very satisfied with the program, and they indicated they would recommend the program to other new nurses. (Cadmus et al., 2016, p. 238)

Support and reward from management are facilitators to implement effective mentorship. Some mentors stated that they should be valued and recognized for their expertise.

One preceptor stated “We should be treated as though we are valued;” another nurse stated, “We should be recognized for our expertise.” (DeCicco, 2008, p. 21)

A traditional workplace hierarchy and an unfettered sense of superiority may lead to mentees being hostile to mentors of the same rank. They distrusted or disobeyed mentors in the same work position, for this reason, making them reluctant to learn from their mentors.

RN nursing students may not be expecting to be supervised by ENs, especially if they are already ENs themselves. Old hierarchies and unchecked feelings of superiority may occur, and ENs can thus be on the receiving end of biases, and potential hostility. (Ryan & McAllister, 2017, p. 5)

In order to establish confidence in the evidence produced, the ConQual approach (Munn et al., 2014) was used to assess confidence in the synthesized findings. The ConQual summary of findings is shown in Appendix S6.

## 4 DISCUSSION

This meta‐synthesis identified eight qualitative studies from diverse countries on nursing home mentorship programmes in a global context. The review by systematically synthesizing qualitative studies has contributed to new knowledge via the three themes: mentor capability, opportunity in the mentorship programmes and motivation in the mentorship programmes. These themes reveal crucial factors that promote or hinder the implementation of mentorship programmes in a nursing home setting. Synthesized findings are rarely reported in individual studies. This systematic review is timely, considering that the demand for mentorship programmes is high, in the context of a lack of staff education and training that could improve staff recruitment and retention, with the ultimate goal of providing high‐quality care for residents in a care setting (Ryan & McAllister, 2017).

Differences exist between hospitals and nursing homes mentorship programmes. Compared to hospitals, which have many available qualified mentors, not all nursing homes are able to offer a mentor for each mentee. Also, the turnover rate of nurses in nursing homes is higher than in hospitals, leading to the possibility that some mentors in nursing homes may leave during a mentorship programme intervention (Bratt & Gautun, 2018). Moreover, there is a relatively sound management and training system for mentors and mentees in hospitals, while mentors in nursing homes may not have the same learning opportunities to improve their capabilities, due to limited institutional funding for staff development (Ko, Wagner, & Spetz, 2018). On the other hand, compared to the busy hospital working environment, the slower pace of nursing home care can provide mentees with sufficient time to learn (Husebo et al., 2018). Mentors appointed in a nursing home are more likely to consider their intentions and willingness to be a mentor, as in this work environment they have a supervisory role for unlicensed staff, compared to their counterparts in hospitals (Cummings et al., 2014).

There are also some similarities in implementing effective mentorship programmes in nursing homes and hospitals. For example, there are broadly similar requirements for the appointment of mentors. Mentors play an educational and leadership role and are required to have rich work experiences, a willingness to share knowledge, and an enthusiasm for teaching and for the success of mentees (Burgess, van Diggele, & Mellis, 2018). The scope of nurses’ work beyond its clinical nature or the performance of non‐nursing tasks, such as teaching, adds complexity to the learning process in both hospitals and nursing homes (Montayre & Montayre, 2017). In addition, there are similar barriers to implementing effective mentorship programmes, for example conflicts of interest between mentors and mentees; an imbalance of power; poor communication; and lack of trust and support (Eller, Lev, & Feurer, 2014). This systematic review mainly focuses on the enablers and potential barriers that impact effective mentorship programmes in nursing homes from the three aspects as indicated in the findings.

Mentor capability is one of the factors affecting the successful implementation of a mentorship programme. Selecting suitable mentors is crucial to effective mentorship, as unqualified mentors are associated with mentee‐mentor conflicts. The qualitative synthesis showed that mentors are mainly assigned based on their specific characteristics, seniority and abilities. This is consistent with other studies (Geraci & Thigpen, 2017; L'Ecuyer, Hyde, & Shatto, 2018; Sambunjak, Straus, & Marusic, 2010). For example, most enrolled nurses (ENs) receive lower levels of education than advanced practice nurses (APNs) (Endacott et al., 2018; Harbman et al., 2017), but the work of ENs is increasingly complex when they provide direct care to residents and are involved in an educational role (Gibson & Heartfield, 2003). ENs can also be chosen as mentors. In addition, improving mentors capabilities through regular education and training is one way to enable and sustain the implementation of these types of programmes. On the basis of Sen's capability theory, Nordenfelt pointed out that internal capabilities (e.g. occupational skill) are crucial and dynamic, requiring training in order to develop (Tengland, 2016). Many nurses with a high educational background, such as APNs, have acquired relevant knowledge and skills, but lower‐level employees, such as nursing assistants, may need additional training to improve their capabilities to guide mentees. In addition, identifying the mentor's role—as advisor, guide, and leader—is helpful in enabling effective mentorship. These roles contribute to a mentor's acquisition of further skills, such as facilitation and counselling, which will support them in their role as a mentor (Schroyer et al., 2016). But if the mentor has a supervisory role for mentees, there may be potential conflicts of interest and a reluctance on the part of mentees to share their problems or challenges. This situation is more likely to occur in resource‐poor nursing homes where the availability of mentor is limited (Burgess et al., 2018).

It has been found that challenges mainly exist in the realm of opportunity. The review has shown that suitable mentor matching is vital in promoting staff participation. If mentees are assigned to a mentor they do not feel comfortable with, it could result in a negative influence (Rohatinsky & Jahner, 2016). It is suggested to popularize the method of using personality questionnaires to help in appropriate matching. The finding is consistent with other studies (Burr et al., 2011; Gisbert, 2017). A positive relationship between mentor and mentee can be helpful in providing acceptance, thereby reducing mentees’ anxiety by emphasizing their value (Alisic, Boet, Sutherland, & Bould, 2016). The lack of defined mentor accountabilities is a barrier to implementing the mentorship programmes, as role ambiguity shifts participant and mentor focus away from programme objectives (Sheppard‐Law, Curtis, Bancroft, Smith, & Fernandez, 2018). Mentors who are seldom available is another serious obstacle that may lead to long‐term mentor stress, and insufficient supervision and training for mentees (Chen & Lou, 2014). High staff turnover rates can worsen the shortage of mentors and contribute to the disruption of mentorship programmes. While turnover and short mentors in nursing homes are not new, this review shows how these issues can interfere with effective mentorship geared towards improving care quality (Husebo et al., 2018).

Recognizing motivation in mentorship programmes is very important in promoting successful programme implementation. Staff proactivity in a mentorship programme can facilitate their participation and change their behaviour (Mills et al., 2019). In addition, the intervention effectiveness of mentorship programmes is related to reward mechanisms. There are some tangible or intangible resources to reward staff in the programmes, for example public praise, gifts. Mentor role modelling can encourage others to learn and make positive behavioural changes (Schoonbeek & Henderson, 2011; Vinales, 2015). Findings from this review highlight that traditional hierarchy is a barrier for mentees to listen to a mentor with the same job title. If a mentee is no longer following the mentor, or if a mentee questions the mentor's decisions due to hierarchy, it may hinder an effective mentorship (Ryan & McAllister, 2017). It is suggested that management should give mentors appropriate power to conduct training programmes. Management also needs to be aware that inherent imbalances in power can lead to dysfunctional behaviour (Rauen et al., 2017).

It is well‐known that a meta‐synthesis is a reinterpretation of others’ interpretations with a number of advantages and limitations. To the best of our knowledge, this review is the first in this area. The inclusion of rigorous qualitative studies ensures a rich yet focused data set. The review includes studies not limited to the English language and published year to ensure adequate literature. The potential limitations of the review include the fact that the review may have missed some papers from an indexed search and grey papers. The evidence in this review arose primarily from nursing staff, with no views from care recipients, family members or managers in nursing homes, which may have resulted in limitations. Also, as all of the studies were conducted in developed Western cultures, the findings may not be applicable elsewhere. The included literature is primarily considered to have low dependability due to the evidence types.

## 5 CONCLUSION

The meta‐synthesis has provided synthesized qualitative evidence that can guide the design, implementation and revise of mentorship programmes in nursing homes. Three themes identified in the systematic review are as follows: mentor capability, opportunity in the mentorship programmes and motivation in the mentorship programmes. These themes can help identify what influences effective mentorship programmes. In addition, more high quality quantitative and qualitative papers are required to establish evidence as to how mentorship programmes delivered in nursing homes can successfully be implemented.

## 6 Implications for research

Recommendations for practice arising from the review are provided in Appendix S7 and, as per guidelines from the Joanna Briggs Institute, have been assigned a Grade of Recommendation (Petrisor & Bhandari, 2007). Grade A is a strong recommendation, whereas Grade B is a weaker recommendation. Research recommendations are provided below. To strengthen the evidence on implementing mentorship programmes in nursing homes, it is necessary to develop new research methods and approaches for mentoring, with a focus on mentoring processes, conditions, consequences and determinants in a broader context.

## ETHICAL APPROVAL

Ethical approval was not required for this paper.
