# Supplementary material for: Nursing home staff experiences of implementing mentorship programmes: A systematic review and qualitative meta‐synthesis
Source: J Nurs Manag. 2020 Feb 3;28(2):188–98. doi: 10.1111/jonm.12876 (PMC7328728; doi:10.1111/jonm.12876)
Supplement: Supplementary file 1 [file JONM-28-188-s001.docx]

Appendix Ⅰ

Search strategies used for finding qualitative research articles about setting up clinical mentors in nursing home. Number of retrieved articles is given in the right-hand column.

**CINAHL (EbscoHOST)**

| S1 | (MH "Stakeholder Participation") | 343 |
| --- | --- | --- |
| S2 | (MH " Case Managers ") | 3453 |
| S3 | (MH " Nurse Administrators ") | 7641 |
| S4 | (MH " Nurses ") | 57993 |
| S5 | (MH " Nursing Assistants ") | 7619 |
| S6 | (MH " Nursing Home Patients ") | 12162 |
| S7 | S1 OR S2 OR S3 OR S4 OR S5 OR S6 | 87734 |
| S8 | AB((“Care aide*” or “Care Attendant*” or “Care Guide*” or “paid caregiver*” or “Certified Nursing Assistant*” ) OR ( “Direct care worker*” or “Geriatric Health Aide*” ) OR (“Health Care Assistant*” or “Institutional Aide*” or “Medical Assistant*” ) OR ( “Nurs* aide*” or “Nurs* assistant*” or “Nurs* home aide*” or “Personal Care Attendant*” )) | 3794 |
| S9 | AB(((elderly or senior* or geriatric or veteran*) N3 institution* ) OR ( (elderly or senior* or geriatric or veteran*) N3 home* ) OR ( (elderly or senior* or geriatric or veteran*) N3 facilit* ) OR ( (elderly or senior* or geriatric or veteran*) N3 unit* ) OR ( (elderly or senior* or geriatric or veteran*) N3 center* )) | 8577 |
| S10 | S7 OR S8 OR S9 | 12294 |
| S11 | AB(homes for the aged or skilled nursing facilities or (nursing adj (home* or center* or centre*) or residence* or residential or long term care or facilit* or institution*) | 356187 |
| S12 | TX (mentor* or leader* or champion* or monitor* or tutors or preceptor or apprenticeship or teach* or train* or mentee) | 1215879 |
| S13 | S10 AND S11 AND S12  Limiters‐Clinical Queries: Qualitative‐High Sensitivity | 896 |

**Medline (OVID) search strategy**

MEDLINE In‐Process & Other Non‐Indexed Citations and Ovid MEDLINE

| 1 | *Mentors/ed [Education] | 280 |
| --- | --- | --- |
| 2 | (mentor* or leader* or champion* or monitor* or tutors or preceptor or apprenticeship or mentee).tw. | 814121 |
| 3 | or/1-2 | 814193 |
| 4 | exp Homes for the Aged/ | 13195 |
| 5 | exp Nursing Homes/ | 37267 |
| 6 | (nursing adj (home* or center* or centre*) or residence* or residential or long term care or facilit* or institution*).tw. | 930846 |
| 7 | or/5-6 | 946091 |
| 8 | Stakeholder Participation/ or Case Managers/ or Nurse Administrators/ or Nurses/ or Nursing Assistants/ or Nursing Staff/ or Nursing Home Patients/ | 72487 |
| 9 | (Care aide* or Care Attendant* or Care Guide* or caregiver* or Certified Nursing Assistant* or CNA* or Direct care worker* or Geriatric Health Aide* or Health Care Assistant* or Institutional Aide* or Medical Assistant* or Nursing aide* or Nursing assistant* or Nursing attendant* or Nursing home aide*).tw. | 62854 |
| 10 | or/8-9 | 133402 |
| 11 | 3 and 7 and 10 | 1806 |
| 12 | Limit 12 to Qualitative(maximize Sensitivity) | 1463 |

**Embase（OVID）**

| 1 | interview:.tw. | 404287 |
| --- | --- | --- |
| 2 | qualitative.tw. | 241989 |
| 3 | exp health care organization/ | 1423327 |
| 4 | 1 or 2 or 3 | 1918475 |
| 5 | *Mentors/ | 565 |
| 6 | (mentor* or leader* or champion* or monitor* or tutors or preceptor or apprenticeship or mentee).tw. | 1010406 |
| 7 | 5 or 6 | 1010476 |
| 8 | exp home for the aged/ | 5894 |
| 9 | exp nursing home/ | 33832 |
| 10 | (nursing adj (home* or center* or centre*) or residence* or residential or long term care or facilit* or institution*).tw. | 1222920 |
| 11 | 8 or 9 or 10 | 1232988 |
| 12 | Stakeholder Participation/ or Case Managers/ or Nurse Administrators/ or Nurses/ or Nursing Assistants/ or Nursing Staff/ or Nursing Home Patients/ | 110046 |
| 13 | (Care aide* or Care Attendant* or Care Guide* or caregiver* or Certified Nursing Assistant* or CNA* or Direct care worker* or Geriatric Health Aide* or Health Care Assistant* or Institutional Aide* or Medical Assistant* or Nursing aide* or Nursing assistant* or Nursing attendant* or Nursing home aide*).tw. | 88134 |
| 14 | 12 or 13 | 194883 |
| 15 | 4 and 7 and 11 and 14 | 2037 |

**Scopus**

| 1 | ALL(mentor* or leader* or champion* or monitor* or tutors or preceptor or apprenticeship or mentee) | 5800797 |
| --- | --- | --- |
| 2 | ABS ( ( "Stakeholder Participation" ) OR ( "Case Managers" ) OR ( "Nurse Administrators" ) OR ( "Nurses" ) OR ( "Nursing Assistants" ) OR ( "Nursing Staff " ) OR ( "Nursing Home Patients" ) ) | 239524 |
| 3 | ABS(“Care aide*” or “Care Attendant*” or “Care Guide*” or “paid caregiver*” or “Certified Nursing Assistant*” or CNA* ) OR ( “Direct care worker*” or “Geriatric Health Aide*” ) OR (“Health Care Assistant*” or “Institutional Aide*” or “Medical Assistant*” ) OR ( “Nurs* aide*” or “Nurs* assistant*” or “Nurs* home aide*” or “Personal Care Attendant*” ) | 26019 |
| 4 | ABS((elderly or senior* or geriatric or veteran*) N3 institution* ) OR ( (elderly or senior* or geriatric or veteran*) N3 home* ) OR ( (elderly or senior* or geriatric or veteran*) N3 facilit* ) OR ( (elderly or senior* or geriatric or veteran*) N3 unit* ) OR ( (elderly or senior* or geriatric or veteran*) N3 center* ) OR ( (elderly or senior* or geriatric or veteran*) N3 centre*) | 3820 |
| 5 | or/2-4 | 263008 |
| 6 | TITLE-ABS-KEY("homes for the aged" or "nursing home*" or "nursing center*" or "nursing centre*" or residence* or residential or "long term care" or facilit* or institution*or "Geriatric Nursing" ) | 3974 |
| 7 | 1 AND 5 AND 6 | 245 |
| 8 | TITLE-ABS-KEY(qualitative OR ethnograph $ OR phenomenology $ OR “grounded theory” OR hermeneutic$ OR “experience$” OR narrative$ OR “action research” OR observation$ OR “focus group$” OR interview$ OR “mixed method” OR “multimethod”) | 4616307 |
| 9 | 7 AND 8 | 121 |

**Web of science**

| 1 | \|  \| TS=( mentor* or leader* or champion* or monitor* or tutors or preceptor or apprenticeship or mentee ) \| \| --- \| --- \| | 1955799 |
| --- | --- | --- | --- | --- |
| 2 | \|  \| AB=(("Stakeholder Participation") OR ("Case Managers") OR ("Nurse Administrators") OR ("Nurses") OR ("Nursing Assistants") OR ("Nursing Staff " ) OR ("Nursing Home Patients" )) \| \| --- \| --- \| | 253746 |
| 3 | AB=(“Care aide*” or “Care Attendant*” or “Care Guide*” or “paid caregiver*” or “Certified Nursing Assistant*” or CNA* OR “Direct care worker*” or “Geriatric Health Aide*” OR “Health Care Assistant*” or “Institutional Aide*” or “Medical Assistant*” OR “Nurs* aide*” or “Nurs* assistant*” or “Nurs* home aide*” or “Personal Care Attendant*” ) | 15302 |
| 4 | \|  \| AB=((elderly or senior* or geriatric or veteran*) N3 institution* OR ( (elderly or senior* or geriatric or veteran*) N3 home* ) OR ( (elderly or senior* or geriatric or veteran*) N3 facilit* ) OR ( (elderly or senior* or geriatric or veteran*) N3 unit* ) OR ( (elderly or senior* or geriatric or veteran*) N3 center* ) OR ( (elderly or senior* or geriatric or veteran*) N3 centre* )) \| \| --- \| --- \| | 31 |
| 5 | 2 or 3 or 4 | 262711 |
| 6 | AB= ("homes for the aged" or "nursing home*" or "nursing center*" or "nursing centre*" or residence* or residential or "long term care" or facilit* or institution*or "Geriatric Nursing" ) | 1466893 |
| 7 | 1 AND 5 AND 6 | 4819 |
| 8 | AB=(qualitative OR ethnograph $ OR phenomenology $ OR “grounded theory” OR hermeneutic$ OR “experience$” OR narrative$ OR “action research” OR observation$ OR “focus group$” OR interview$ OR “mixed method” OR “multimethod” ) | 2776639 |
| 9 | 7 and 8 | 1809 |

**PsycINFO**

| 1 | TX (mentor* or leader* or champion* or monitor* or tutors or preceptor or apprenticeship or mentee) | 307515 |
| --- | --- | --- |
| 2 | TX（Stakeholder Participation/ or Case Managers/ or Nurse Administrators/ or Nurses/ or Nursing Assistants/ or Nursing Staff/ or Nursing Home Patients/） | 73034 |
| 3 | AB(Care aide* or Care Attendant* or Care Guide* or caregiver* or Certified Nursing Assistant* or CNA* or Direct care worker* or Geriatric Health Aide* or Health Care Assistant* or Institutional Aide* or Medical Assistant* or Nursing aide* or Nursing assistant* or Nursing attendant* or Nursing home aide*) | 10188 |
| 4 | or/2-3 | 80852 |
| 5 | AB("homes for the aged" or "nursing home*" or "nursing center*" or "nursing centre*" or residence* or residential or "long term care" or facilit* or institution*or "Geriatric Nursing" ) | 43997 |
| 6 | 1 AND 4 AND 5 | 3158 |
| 7 | AB(qualitative OR ethnograph $ OR phenomenology $ OR “grounded theory” OR hermeneutic$ OR “experience$” OR narrative$ OR “action research” OR observation$ OR “focus group$” OR interview$ OR “mixed method” OR “multimethod”) | 137348 |
| 8 | 6 AND 7 | 1074 |

TOTAL FOUND: 7397
