# Supplementary material for: Nursing home staff experiences of implementing mentorship programmes: A systematic review and qualitative meta‐synthesis
Source: J Nurs Manag. 2020 Feb 3;28(2):188–98. doi: 10.1111/jonm.12876 (PMC7328728; doi:10.1111/jonm.12876)
Supplement: Supplementary file 2 [file JONM-28-188-s002.docx]

Appendix Ⅱ：List of excluded studies

|  | Study | Reasons for exclusion |
| --- | --- | --- |
| 1 | O'Neil, K. (2008). Preceptorship in home care. Home Healthc Nurse, 26(9), 525-532. | No evidence of ethics approval and did not present statement locating the researcher culturally or theoretically and the influence of the researcher on the research. |
| 2 | Scalzi, C. C., Evans, L. K., Barstow, A., & Hostvedt, K. (2006). Barriers and enablers to changing organizational culture in nursing homes. Nurs Adm Q, 30(4), 368-372. | No evidence of ethics approval and date that represents participant's views; unable to determine where conclusions are drawn from; did not present statement locating the researcher culturally or theoretically and the influence of the researcher on the research. |
| 3 | O'Brien, J., Ringland, M., & Wilson, S. (2010). Advancing nursing leadership in long-term care. Nurs Leadersh (Tor Ont), 23 Spec No 2010, 75-89. | Participant's views are rare; unable to determine where conclusions are drawn from; no evidence of ethics approval and did not present statement locating the researcher culturally or theoretically and the influence of the researcher on the research. |
| 4 | Aaron, C. S. (2011). The positive impact of preceptors on recruitment and retention of RNs in long-term care. J Gerontol Nurs, 37(4), 48-54. | Participant's views are rare; no evidence of ethics approval and did not present statement locating the researcher culturally and the influence of the researcher on the research. |
