# Supplementary material for: Nursing home staff experiences of implementing mentorship programmes: A systematic review and qualitative meta‐synthesis
Source: J Nurs Manag. 2020 Feb 3;28(2):188–98. doi: 10.1111/jonm.12876 (PMC7328728; doi:10.1111/jonm.12876)
Supplement: Supplementary file 3 [file JONM-28-188-s003.docx]

Appendix Ⅲ: QARI data extraction of included studies

| Author(year) | Methodology | Method | Phenomena of interest | Setting | Geographical | Cultural | Participants | Data analysis | Author^’^s conclusion | Reviewer^’^s comments |
| --- | --- | --- | --- | --- | --- | --- | --- | --- | --- | --- |
| Kaasalainen, S et al.(2015) | Case study | Participant observation, Diaries recording, Interviews and Focus groups | Explore the role of a CNS and an NP as change champions during the pain protocol implementation process. | Two LTC facilities in southern Ontario, Canada | Southern Ontario, Canada | Canada | Various members of the interdisciplinary teams responsible for implementing the pain protocol. | Thematic content analysis, NVIVO | The CNS and NP are ideal champions to implement pain management and quality improvement. | Show the role of CNS and NP as change champions. And describes the obstacles and facilitators to mentoring. |
| Kaasalainen, S et al.(2016) | Mixed methods | Interviews and Focus groups | To evaluate the effectiveness of implementing a nurse practitioner-led, inter-professional pain management team in LTC in improving (a) pain-related resident outcomes; clinical practice behaviours and quality of pain medication prescribing practices. | Six LTC facilities in Canada | Canada | Canada | Personal support workers (care aides), licensed nurses and NPs at each of the full intervention sites. | Thematic content analysis | NPs play an important role in facilitating IP collaboration, implementing an NP-led Pain Team can significantly improve clinical practice behaviors of LTC staff and reduce resident pain. | Show the importance and personal attribute of the champions. |
| Ploeg et al.(2010) | Mixed methods | Telephone interviews | How nursing best practice champions influence the diffusion of Best Practice Guideline recommendations. | Long‐term care (LTC) in Canada | Canada | Canada | 7 champions from LTC | Categorization of findings | The nurse champions have a multidimensional role, which can help service organization and nursing profession implement evidence-based practices. | Describe the role of nursing best practice champions. |
| Aubry et al.(2012) | Qualitative study | In-depth semi-structured interviews | The process by which new nursing assistants are integrated into LTCOs | Two long term care centers | Quebec, Canada | Canada | 23 nursing assistants | Semi-inductive analysis based on Crozier’s framework | Expanding the role of nursing assistant appears to be more appropriate than destroying the various structures that jointly hold up their current service network. | Present various ways to guide the mentees. |
| Cadmus et al.(2016) | Mixed methods | Voluntary focus groups | Views and experiences of Preceptors and Nurse Residents about implementing New Nurse Residency program in LTC | Long term care centers | New Jersey | The United States | 6 the preceptors and 8 the nurse residents | Not clear | There are many opportunities for new RNs to expand their practice. A residency program for the new nurse is one strategy to help improve recruitment and retention. | Identify the characteristics of mentors and challenges to implement effective mentorship. |
| Rohatinsky and Jahner(2016) | Interpretive description methodology | Semi-structured interview | The impact of mentorship on new staffs | One rural care facility | Saskatchewan, Canada | Canada | Seven female, all were RNs or LPNs. | Constant comparative analysis | Mentorship is a vital component to personal and professional success of new employees in rural areas | Present barriers and strategies to develop mentorship program. And shows the role of mentors. |
| Ryan and McAllister(2017) | Qualitative | Semi-structured group interviews | Enrolled Nurses’ experiences learning the nurse preceptor role | Two regional care facilities | Australia | Australia | Fifteen ENs participated. Two were males and thirteen females | Thematic analysis, Van Manen’s (1997) three step method | EN preceptors value and benefit from engagement in learning experiences. Learning that is accessible, solution-focused, and includes role modelling of critical and creative thinking was appreciated. | Identify barriers to develop preceptorship program. And describe the experiences of mentors in guiding mentees. |
| DeCicco(2008) | Qualitative | Focus groups, key informant interviews, and a workflow analysis | Factors that influence preceptorship | Not clear | Ontario | Canada | 27 employees | Content analysis | Preceptorship is fundamental within today’s community nursing practice | Understand the benefits of implementing mentorship program and related factors to promote the program. |
