# Supplementary material for: Nursing home staff experiences of implementing mentorship programmes: A systematic review and qualitative meta‐synthesis
Source: J Nurs Manag. 2020 Feb 3;28(2):188–98. doi: 10.1111/jonm.12876 (PMC7328728; doi:10.1111/jonm.12876)
Supplement: Supplementary file 4 [file JONM-28-188-s004.docx]

**Appendix Ⅳ: Finding and illustrations**

Kaasalainen, S. , Ploeg, J. , Donald, F. , Coker, E. , Brazil, K. , & Martin-Misener, R. , et al. (2015). Positioning clinical nurse specialists and nurse practitioners as change champions to implement a pain protocol in long-term care. Pain Management Nursing, 16(2), 78-88.

| Finding | Illustration from study | Evidence |
| --- | --- | --- |
| The CNS and NP as change champions provided one-on-one outreach to educate staff. | [The NP] was like the library, to help us understand the pain protocol a little better, the pain assessment a little bit better. Understanding the resident, with the different diagnoses, the reasons for having different pain.or maybe expressing pain in a different way. (p.82) | Unequivocal |
| The role of CNS as change champions. | CNS met with a clinical leader to discuss pain management of residents on unit.Talked to nurses about using a standardized pain assessment tool. Distributed the pain protocol resource binders and  binders for interdisciplinary staff members; CNS  informed the staff in each clinical area of the following related to the protocol: purpose of the study, and contents of the protocol resource binder, including brief summaries of the journal/research articles found in the binder. (p.82-83) | Unequivocal |
| Assisting staffs in solving problems. | Could the resident’s behavior be related to pain? If the staff came to me questioning a resident’s behavior, we would attempt to determine if it was pain-related together. (p.83) | Unequivocal |
| Organizing and Facilitating Scheduled Educational Sessions to facilitate success. | The NP developed and facilitated interactive educational in-service for team (RNs, PSWs, physiotherapist) re: types of pain. (p.83) | Unequivocal |
| The CNS and NP provided reminders and prompts to Staff by educational poster board or electronic system. | I think putting those [pain assessment tools] onto a  computerized version was just a lot easier for the staff too as a reminder to automatically do that. And it just made them think of it, too. It just made a lot of people more aware of pain and what it looked like. (p.83) | Unequivocal |
| The change champions prompt practice and sustain change by using audit and feedback. | I [NP] reviewed and audited charts to determine if staff were completing the pain protocol appropriately. I [NP] asked staff to add more details or further explain in their charting about resident pain. (p.84) | Unequivocal |
| The NP was engaged in providing direct care. | The NP went through entire medication list (explaining why patient is on each med, dosage, and possible side effects). Example of pain medications: fentanyl patch 100 mcg (3 patches) Q72h, oxycontin CR 40 mg Q12h, oxycocet 5/325 2 tabs po Q4h prn. (p.84) | Unequivocal |
| The CNS and NP as change champions had strong communication and interpersonal skills, were highly respected within their respective organizations, and were viewed as clinical opinion leaders by staff. | I think that [NPs] are able to display a higher level of  knowledge and understanding and so they gain the respect of the nurses who see them that way and not necessarily just another pair of hands. So I think because [the registered nursing staff] have road tested and can see the value of what [NPs] have to offer that I think that has probably helped as well. So [the registered nursing staff] will automatically know if something is not really clear to them say well maybe we need to talk to [the NPs], maybe they need to come and see the resident. (p.85) | Unequivocal |
| Creating positive relationships with staff to  facilitate practice changes. | It really is being there, being around, having your ears opened for what is happening as well as responding when people come to you. But it’s a constant presence, right, you don’t just sort of drop something one day and say oh,here you go. Some people you can, some people just need the information and they are able to sort of run with that.And other folks it’s a little more of a struggle, it’s a companion you know. In our particular model we have the nurses doing a lot of tasks, your mind isn’t free to think beyond the medication cart. So just having that extra mind there to say, ‘‘Why don’t you try this?’’ ‘‘Have you thought about that?’’ Or ‘‘Can I do this for you?’’ It can’t just be a parachute  sort of thing. It’s this constant daily walk together, I think,that makes the difference. (p.85) | Unequivocal |
| CNS and NP can provide diversified  support to educate staff. | Having CNS and NP support for educating staff about pain assessment and management. (p.85) | credible |
| Support from administration as facilitators for CNSs and NPs as Change Champions. | Facilitators to implementing the pain protocol included having support from administration for the project, and having CNS or NP support for educating staff about pain assessment and management and use of the pain protocol. (p.85) | credible |

Kaasalainen, S. , Wickson-Griffiths, A. , Akhtar-Danesh, N. , Brazil, K. , Donald, F. , & Martin-Misener, R. , et al. (2016). The effectiveness of a nurse practitioner-led pain management team in long-term care: a mixed methods study. International Journal of Nursing Studies, 62, 156-167.

| Finding | Illustration from study | Evidence |
| --- | --- | --- |
| The personal attributes and approaches to care of the NPs are regarded highly. | the staff highly regarded the personal attributes of the NPs, which included being approachable, dependable, knowledgeable and having clinical  expertise. They characterized the NPs as being effective in their roles and drivers for positive change. (p.162) | credible |
| Positive outcomes from having the NP on the team are highlighted. | The staff at both intervention sites identified that NP  involvement improved team collaboration as well as knowledge about pain assessment and management through education. (p.162) | credible |
| Benefits of the pain team | The staff at the intervention sites highlighted benefits of the Pain Team, specifically by contributing to staff education, using best practices as well as promoting team collaboration, communication and autonomy. (p.163) | credible |
| Limited NP and the lines of communication among staff as barriers to pain management and effective pain team. | They also recognized several factors that were impeding efficacy of the Pain Team and pain management including: the large number of staff at the home in need of pain education, the lines of communication among staff, and the limited NP availability in the LTC home. (p.164) | credible |

Ploeg, J., Skelly, J., Rowan, M., Edwards, N., Davies, B., Grinspun, D. Downey, A. (2010). The Role of Nursing Best Practice Champions in Diffusing Practice Guidelines: A Mixed Methods Study. Worldviews Evid Based Nurs, 7(4), 238-251.

| Finding | Illustration from study | Evidence |
| --- | --- | --- |
| Education and awareness | In many cases education was described as ongoing  and designed to “continuously reinforce . . . best practices”  (B111, LTC)（p.245） | Unequivocal |
| Education strategies | Most efforts were directed at organizing or delivering face-to-face, group education sessions,  workshops, or presentations to educate staff. Several champions provided electronic or video-based learning, for example, through an“interactive web-based learning CD (with) links to RNAO and other wound care sites” (A109, Acute)（p.244） | Unequivocal |
| Feeling passionate about the work. | “I’m one of the clinical leaders. . . . That’s in my job description and I guess that’s one of my passions . . . to ensure that the residents in our facility are being cared for with best practices, and evidence-based  practice” (B110, LTC)（p.245） | Unequivocal |
| Champions as persuasive practice leaders. | Champions worked with staff, peers, other professionals, and senior managers on a variety  of groups and committees to persuasively advocate for evidence-based practice through BPG utilization. （p.245） | credible |
| Interacting with team members. | Champions indicated that although their team work involved mostly nurses, they also interacted with a host of other interdisciplinary team members. “anything relating to OT (Occupational Therapy) or Physio (Physiotherapy), our members of the team would then take that back to those people and discuss things that were going on. . . . We spoke with [nurses] on the floor” (P.246) | Unequivocal |
| Exploring, auditing, and monitoring best practices. | “I would go to the web . . . and start looking for best practice guidelines and what can we do to incorporate that into our practice. It’s based on what the population need is at the time” (p.246) | Unequivocal |

Aubry, F. , Etheridge, F. , & Couturier, Y. . (2013). Facilitating change among nursing assistants in long term care. Online J Issues Nurs, 18(1), 8.

| Finding | Illustration from study | Evidence |
| --- | --- | --- |
| The work patterns/behaviors they learned during their training did not coincide with the limited time they were given to perform their daily activities. | “during our training, we are told to be careful,to take whatever time we need…that is why it is hard when we start working in the organization.…” | Unequivocal |
| Integrating new recruits into LTCOs to manage heavy workloads. | “I’m in charge of the orientation of new recruits…We look at results working ; we see how they are when they start; we see what happens. It takes a certain pace… And we help them to keep the pace because they don’t learn that during their training.” | Unequivocal |
| Transmission of informal work strategies to enhance the work of nursing care. | “I tell those who come here for meal times to feed two at a time…I once served three at once. You put two on either side of you and feed them alternately to avoid losing any time.” Such strategies, created and used by nursing assistants and transmitted from experienced nursing assistants to new recruits, illustrate the incredible resourcefulness of these nursing assistants as they tried to meet their workload requirements. | Unequivocal |

Cadmus, E. , Salmond, S. W. , Hassler, L. J. , Black, K. , & Bohnarczyk, N. . (2016). Creating a long-term care new nurse residency model. Journal of Continuing Education in Nursing, 47(5), 234.

| Finding | Illustration from study | Evidence |
| --- | --- | --- |
| Taking pride in sharing knowledge. | The preceptors noted they had an increase in personal pride in being able to share knowledge. They also identified they had been exposed to new methods of learning such as the simulation exercises.（p.238） | credible |
| The biggest hurdles identified by preceptors were time and staffing issues. | The biggest hurdles identified by preceptors were time and staffing issues they needed to contend with during the course of the program. （p.238） | credible |
| The preceptor program Improved residents' satisfaction. | The nurse residents were very satisfied with the program, and they indicated they would recommend the program to other new nurses. （p.238） | credible |
| Improve nurse residents' satisfaction. | Several of the nurse residents identified that the program made them want to stay（p.238） | credible |
| Improved staff s' job satisfaction. | through this program and the support provided by the preceptor, facility, and faculty, they learned the important role they played in caring for older adults in the LTC setting, which improved their job satisfaction. （p.238） | credible |
| Encouraging facilities to select the nurse in the educator role as the preceptor. | To reduce this problem in the second cohort, faculty encouraged facilities to select the nurse in the educator role as the preceptor. The other reason for selecting the educator as preceptor is that the  resources provided could be woven into the orientation and education of their current staff. （p.238） | credible |

Jahner, S. (2016). Supporting nurses' transition to rural healthcare environments through mentorship. Rural & Remote Health, 16(1), 3637.

| Finding | Illustration from study | Evidence |
| --- | --- | --- |
| The role of mentorship in rural healthcare environments. | If there was a formal mentorship program, then it would be easier to transition. (Employee B) （p.4） | Unequivocal |
| staffs’ definition of mentorship focused on the development of new staff members. | Mentorship to me is when a seasoned nurse takes a person just entering their career under their wing and just tries to help them with the growing process. (Employee G) （p.4） | Unequivocal |
| mentorships assisted with alleviating feelings of isolation and provided someone with whom to discuss the mixed emotions of the transition  experience. | Sometimes it can be hard to find your way into a small town, [and] tightly knit, staff group. And everybody has their ways, and when you don’t know the ways or you want to try and change things, it can be tough. But with a mentorship, you got that friend there that you can talk to about tough days, and they’ll help you through. (Employee E) （p.4） | Unequivocal |
| Mentors eased the stress of staffs by making independent decision. | … it’s a very different workplace than working in the city. And it can be very scary for them [new staff] because we have one RN, one LPN on a night shift. And we do get some very sick people. (Employee F) （p.5） | Unequivocal |
| Close relationships help foster mentorships. | And they were just so just friendly and embracing of [me]. They just made me feel really appreciated that I was coming to work casual. One of the staff members gave me a hug my first day and she was just so happy that I was here. I think that just that positive environment makes you want to stay. And so that kind of begins the relationship off in a good way.(Employee B) （p.6） | Unequivocal |
| Mentorship gave new employees the confidence and security. | She really knows the community. She knows the people. She knows the dynamics. And without her, I don’t know if I actually would have stayed in that position. (Employee B) （p.7） | Unequivocal |
| Mentorships were beneficial to the mentor. | Actually, I learned a lot about myself, I was fairly new nurse so I was feeling like, ‘am I really doing a good job?’ I learned where my strengths were in my nursing career and even in my interpersonal connections with my other work mates, I learned  and grew just from teaching somebody else. (Employee D) （p.7） | Unequivocal |
| Barrier to mentorship was the lack of staff working at one time. | One LPN and RN would be working together, resulting in limited opportunities for same-profession mentorship on a given shift. （p.7） | Unequivocal |
| A related challenge was the limited selection of available and willing mentors in rural settings to best meet protégé needs. | You don’t have very many people to draw from for support, there are only so many of us that work. Just like if you can’t establish a good friendship with the mentor or the person being mentored, and there’s nowhere else for them to go to, it can be very difficult. (Employee D) （p.7） | Unequivocal |
| Desired characteristics of mentors. | Ask people to be mentors who are truly interested in their job, who have a good work ethic and are a good example for the new nurses coming. People who are non-judgmental and the ones who will sit back and listen, not just be the bossy, chirpy ones who want to control everything, because you really have to let those new nurses develop their art of nursing. (Employee F) （p.7） | Unequivocal |
| The importance of a personality connection between the mentor and protégé. | I knew their personality, I got to know them a bit better. And I think with rural [nursing] you’re going to be working with the same seven RNs all the time. It makes a big difference knowing who you get along with. If I was assigned to somebody who I didn’t feel comfortable with, it would have made a complete difference. (Employee A) （p.8） | Unequivocal |
| Using personality questionnaires to aid in matching. | Participants felt protégés should have input with respect to mentor selection. One newer nurse suggested getting to know staff first before establishing a formal mentorship with an individual.（p.8） | credible |
| Trust needed to be established before the  relationship could be fostered. | An obstacle for mentorship would be if one person in the relationship doesn’t trust the other person with their feelings and their thoughts. (Employee F) （p.8） | Unequivocal |
| Clarifying the relationship and responsibilities between mentor and mentee. | I really got nothing. It was like ‘here’s your mentor’ and that was pretty much it. So it was kind of up to me and my mentor to figure out what our relationship was as opposed to having information. (Employee A) （p.8） | Unequivocal |
| The disapproval of managements. | … it would be either not approved by management or you’d have to pay out of your own pocket to go and attend the workshop. So maybe if you offered [the mentorship workshop in] one central rural area once a year then people can travel to [a more urban rural town] where it’s not far. (Employee A) （p.8） | Unequivocal |

**Ryan, C., & McAllister, M. (2017). Enrolled Nurses' experiences learning the nurse preceptor role: A qualitative evaluation. Collegian, 24(3), 267-273.**

| Finding | Illustration from study | Evidence |
| --- | --- | --- |
| Lack of training of preceptors. | They [other nurses] say to me ‘‘you’ve done the preceptor training the student is yours’’. . .RNs or ENs, it doesn’t matter. Mostly I don’t know what to do with them [the students]. I wish sometimes someone could help me. {Brian}（p.4）  I’ve never been asked or invited to preceptor training at [names hospital]. Why is that? I believe training is so important. Why haven’t I been when we all have students all the time? {Jane}（p.4） | Unequivocal |
| Participants identified four main areas for additional training; feedback, reflective practice, assessing students, and teaching strategies. | ……I need to learn more about giving and receiving feedback. {Louise}  …… I need more training in reflective practice. {Helen}  ……If I could assess students properly that would help me give better feedback. {Donna}（p.4） | Unequivocal |
| Open to embracing opportunities to learn about  clinical teaching. | This is really good! Are we able to look at this at home later . . .or is it just for this session? {Paul}（p.4） | Unequivocal |
| Barriers to engagement with N2E. | Time is the issue here. I don’t have time to show/teach students properly or to do courses. We get no recognition and no paid study leave. {Brian}（p.5） | Unequivocal |
| Understanding the unique experiences of EN  preceptors. | Expecting ENs to preceptor students from other  practice domains may pose an unsafe ethical as well as a personal dilemma for EN preceptors, nursing students and health care consumers. （p.5） | credible |
| RN students' distrust to their mentors. | What do I do when RN students say, ‘‘You’re just an EN! I will not learn anything!’’ I think this website could help. I think I need to learn to convince RN students I have much to teach them. {Shel}（p.5） | Unequivocal |
| The predicament of preceptoring students who preparing to become RNs. | RN nursing students may not be expecting to be supervised by ENs, especially if they are already ENs themselves. Old hierarchies and unchecked feelings of superiority may occur, and ENs can thus be on the receiving end of biases, and potential hostility. （p.5） | credible |

**DeCicco, & Julie. (2008). Developing a preceptorship/mentorship model for home health care nurses. Journal of Community Health Nursing, 25(1), 15-25.**

| Finding | Illustration from study | Evidence |
| --- | --- | --- |
| It is believed that preceptorship supports recruitment and retention within the organization. | One preceptor stated “New hires who feel supported are more likely to stay,” and another stated, “Students who enjoy the preceptorship experience apply for jobs when they graduate.”（p.20） | Unequivocal |
| Preceptors benefit from having more accountability and responsibility, that can lead to advancement within the organization. | Preceptors stated that they “enjoy what they do” and take great pride when a new hire “becomes an asset to the team.” （p.20） | Unequivocal |
| Preceptees benefit from the experience by feeling supported and connected to the organization | One preceptee stated “It made the transition to the community much easier.” （p.20） | Unequivocal |
| The successful preceptorship program is related to the areas of variation in preceptorship processes. | One key informant highlighted the benefits of a more standardized preceptorship program, noting that this would promote “more consistent and efficient practices across the organization.” （p.20） | credible |
| The successful preceptorship program is related to the areas of accountability for the preceptorship program. | The workflow analysis revealed that one of the main challenges with our preceptorship program was a lack of clearly defined accountability. Participants suggested that the new model must clearly identify singular accountability within each service delivery center (SDC) for the new hire and their preceptorship experience. （p.20-21） | Unequivocal |
| The successful preceptorship program is related to the areas of continuity of preceptors. | Preceptees identified that they were not always paired with one consistent preceptor. One new hire stated “I wasn’t really paired with anyone in particular. I think I had three preceptors.” （p.21） | Unequivocal |
| Lacking of trained preceptors | It was also discovered that nurses who had not been through the training process, and who were not particularly interested in being preceptors, were still asked to take on the role due to a lack of trained preceptors within the SDC（p.21） | credible |
| The successful preceptorship program is related to the areas of rewarding and recognition for preceptors. | One preceptor stated“We should be treated as though we are valued;” another nurse stated, “We should be recognized for our expertise.” （p.21） | Unequivocal |
| The successful preceptorship program is related to the areas of “protected” time to precept. | One new hire stated, “They [the preceptors]  have to rush from one client to the next, and when they finally do have time to talk, it is at the end of a busy day when both [nurses] are tired.” （p.21） | Unequivocal |
| Preceptorship was a worthwhile investment and that preceptors are a valuable resource. | “There is a great deal of learning and growth in  being a preceptor”（p.21） | Unequivocal |
| Identifying the nurse educator or clinical resource nurse (CRN) as the responsible person for coordinating the preceptorship program. | The CRN is now responsible for monitoring and evaluating new hires and student orientation experiences, which includes matching preceptees with preceptors and ensuring the completeness of the preceptee’s learning plan. Furthermore, the CRN is also responsible for educating and training the preceptors and, although it was occurring informally in the previous model, the new model formally acknowledges the CRN as the mentor for preceptors, new hires, and students. （p.23） | credible |
