# Supplementary material for: Nursing home staff experiences of implementing mentorship programmes: A systematic review and qualitative meta‐synthesis
Source: J Nurs Manag. 2020 Feb 3;28(2):188–98. doi: 10.1111/jonm.12876 (PMC7328728; doi:10.1111/jonm.12876)
Supplement: Supplementary file 5 [file JONM-28-188-s005.docx]

Appendix Ⅴ: Results of meta-synthesis

| **Finding** | **Category** | **Synthesized findings** |
| --- | --- | --- |
| The CNS and NP as change champions provided one-on-one outreach to educate staff（U） | Selecting mentors | Synthesized findings 1: Mentor capability.  It is important to recognise that mentor capability exerts an impact on the development of mentorship education programmes, as well as on participant experiences. Mentors need training in order to improve their capability, regardless of their original level of education. Choosing appropriate mentors and defining their roles based on their capabilities are also important factors in ensuring mentor capability. |
| The CNS and NP as change champions had strong communication and interpersonal skills, were highly respected within their respective organizations, and were viewed as clinical opinion leaders by staff（U） |  |  |
| The personal attributes and approaches to care of the NPs are regarded highly（C） |  |  |
| Encouraging facilities to select the nurse in the educator role as the preceptor（C） |  |  |
| Desired characteristics of mentors（U） |  |  |
| Identifying the nurse educator or clinical resource nurse (CRN) as the responsible person for coordinating the preceptorship program（C） |  |  |
| The role of CNS as change champions（U） | The role of mentor |  |
| Assisting staffs in solving problems（U） |  |  |
| Organizing and Facilitating Scheduled Educational Sessions to facilitate success（U） |  |  |
| The NP was engaged in providing direct care（U） |  |  |
| Positive outcomes from having the NP on the team are highlighted（C） |  |  |
| Benefits of the pain team（C） |  |  |
| Champions as persuasive practice leaders（C） |  |  |
| Exploring, auditing, and monitoring best practices （U） |  |  |
| Integrating new recruits into LTCOs to manage heavy workloads（U） |  |  |
| Staffs’ definition of mentorship focused on the development of new staff members（U） |  |  |
| Mentorships assisted with alleviating feelings of isolation and provided someone with whom to discuss the mixed emotions of the transition  experience（U） |  |  |
| Mentors eased the stress of staffs by making independent decision（U） |  |  |
| Mentorship gave new employees the confidence and security（U） |  |  |
| It is believed that preceptorship supports recruitment and retention within the organization（U） |  |  |
| Preceptees benefit from the experience by feeling supported and connected to the organization（U） |  |  |
| Education and awareness （U） | Training and education |  |
| Lack of training of preceptors（U） |  |  |
| Participants identified four main areas for additional training; feedback, reflective practice, assessing students, and teaching strategies（U） |  |  |
| Using personality questionnaires to aid in matching （C） | Mentor matching | Synthesized findings 2: Opportunities in the mentorship programme.  It is crucial to note that a successful mentorship programme implementation is mainly associated with participant opportunities to engage in the programme. Appropriate mentor matching is conducive to create trusting relationships, and creating a supportive environment through various positive styles of mentoring can facilitate staff participation in these types of programmes. An awareness of the factors that reduce opportunities to participate in mentorship activities, including a lack of defined accountability, time constraints, and unavailable mentors, is important. |
| Understanding the unique experiences of EN  preceptors（C） |  |  |
| The successful preceptorship program is related to the areas of continuity of preceptors（U） |  |  |
| Creating positive relationships with staff to  facilitate practice changes（U） | Trusting relationships |  |
| Close relationships help foster mentorships（U） |  |  |
| The importance of a personality connection between the mentor and protégé（U） |  |  |
| Trust needed to be established before the  relationship could be fostered（U） |  |  |
| The CNS and NP provided reminders and prompts to Staff by educational poster board or electronic system（U） | Diverse mentoring styles |  |
| The change champions prompt practice and sustain change by using audit and feedback（U） |  |  |
| CNS and NP can provide diversified  support to educate staff（C） |  |  |
| Limited NP and the lines of communication among staff as barriers to pain management and effective pain team（C） |  |  |
| Education strategies（U） |  |  |
| Interacting with team members（U） |  |  |
| Transmission of informal work strategies to enhance the work of nursing care（U） |  |  |
| Clarify the relationship and responsibilities between mentor and mentee（U） | The lack of defined accountability |  |
| Preceptors benefit from having more accountability and responsibility, that can lead to advancement within the organization（U） |  |  |
| The successful preceptorship program is related to the areas of variation in preceptorship processes（C） |  |  |
| The successful preceptorship program is related to the areas of accountability for the preceptorship program（U） |  |  |
| The work patterns/behaviors they learned during their training did not coincide with the limited time they were given to perform their daily activities（U） | Time constraints |  |
| The biggest hurdles identified by preceptors were time and staffing issues（C） |  |  |
| Barriers to engagement with N2E（U） |  |  |
| The successful preceptorship program is related to the areas of “protected” time to precept（U） |  |  |
| Barrier to mentorship was the lack of staff working at one time（U） | Unavailable mentor |  |
| Available and willing mentors in rural settings to best meet protégé needs（U） |  |  |
| Lacking of trained preceptors（C） |  |  |
| Feeling passionate about the work（U） | Proactivity | Synthesized findings 3: Motivation in the mentorship programme.  Nursing staff proactivity can facilitate effective mentorship and motivate staff to engage in their programme. Management support and rewards can also motivate staff and enhance staff engagement. Traditional hierarchy may reduce the motivation of mentees who hold the same position as their mentor. |
| Taking pride in sharing knowledge（C） |  |  |
| The preceptor program Improved residents' satisfaction（C） |  |  |
| Improve nurse residents' satisfaction（C） |  |  |
| Improved staff s' job satisfaction（C） |  |  |
| The approval of mentorship program（U） |  |  |
| Mentorships were beneficial to the mentor（U） |  |  |
| Open to embracing opportunities to learn about  clinical teaching（U） |  |  |
| Preceptorship was a worthwhile investment and that preceptors are a valuable resource（U） |  |  |
| Support from administration as facilitators for CNSs and NPs as Change Champions（C） | Support and reward of management |  |
| The disapproval of managements（U） |  |  |
| The successful preceptorship program is related to the areas of rewarding and recognition for preceptors（U） |  |  |
| RN students' distrust to their EN mentors（U） | Traditional hierarchy |  |
| The predicament of preceptoring students who preparing to become RNs（C） |  |  |
