# Supplementary material for: Nursing home staff experiences of implementing mentorship programmes: A systematic review and qualitative meta‐synthesis
Source: J Nurs Manag. 2020 Feb 3;28(2):188–98. doi: 10.1111/jonm.12876 (PMC7328728; doi:10.1111/jonm.12876)
Supplement: Supplementary file 6 [file JONM-28-188-s006.docx]

Appendix Ⅵ：ConQual summary of findings

| Systematic review title: Nursing home staff experiences of implementing mentorship programmes: a systematic review and qualitative meta-synthesis.  Population: All employee working in a nursing home where a mentorship programme was implemented.  Phenomena of interest: Staff experiences of participating in a nursing home mentorship programme.  Context: The context was nursing home, including residential aged care facilities, long-term care. | | | | |
| --- | --- | --- | --- | --- |
| Synthesised finding | Type of research | Dependability | Credibility | ConQual score |
| **Mentor Capability.**  It is important to recognise that mentor capability exerts an impact on the development of mentorship education programmes, as well as on participant experiences. Mentors need training in order to improve their capability, regardless of their original level of education. Choosing appropriate mentors and defining their roles based on their capabilities are also important factors in ensuring mentor capability. | Qualitative | Downgrade  1 level* | Downgrade  1 level** | Low |
| **Opportunity in the mentorship programme.**  It is crucial to note that a successful mentorship programme implementation is mainly associated with participant opportunities to engage in the programme. Appropriate mentor matching is conducive to create trusting relationships, and creating a supportive environment through various positive styles of mentoring can facilitate staff participation in these types of programmes. An awareness of the factors that reduce opportunities to participate in mentorship activities, including a lack of defined accountability, time constraints, and unavailable mentors, is important. | Qualitative | Downgrade  1 level* | Downgrade  1 level** | Low |
| **Motivation in the mentorship programme.**  Nursing staff proactivity can facilitate effective mentorship and motivate staff to engage in their programme. Management support and rewards can also motivate staff and enhance staff engagement. Traditional hierarchy may reduce the motivation of mentees who hold the same position as their mentor. | Qualitative | Downgrade  1 level* | Downgrade  1 level** | Low |

*Downgraded one level due to common dependability issues across the included primary studies (the majority of studies did not present a statement locating the researcher culturally or theoretically, and there was no acknowledgment of their influence on the research).

**Downgraded one level to a mix of unequivocal and credible findings.
