# Supplementary material for: Nursing home staff experiences of implementing mentorship programmes: A systematic review and qualitative meta‐synthesis
Source: J Nurs Manag. 2020 Feb 3;28(2):188–98. doi: 10.1111/jonm.12876 (PMC7328728; doi:10.1111/jonm.12876)
Supplement: Supplementary file 7 [file JONM-28-188-s007.docx]

Appendix Ⅶ: Recommendation for practice

| Recommendation | Joanna Briggs Institute Grade |
| --- | --- |
| The nursing home should select mentors based on their capabilities and personal attributes. The mentor should be enthusiastic, supportive and approachable, representing their respect for the mentees. | A |
| The nursing home should define the role of mentor clearly, because role ambiguity shift the participants and the mentors focus away from the program objectives. | A |
| The nursing home should provide regular professional training to improve mentors' capability, especially should strengthen reflective practice and leadership training to enhance their professional socialization. | A |
| Online communication and face-to-face training should be combined to provide more educational resources. | A |
| The nursing home should conduct appropriate mentor matching by personality questionnaire, which can facilitate effective mentorship. If mentors were assigned, rather than selected by the mentee, the mentorship relationship would be broken easily. | A |
| All members of organization should be encouraged to establish a trustful and positive relationship in order to facilitate effective mentorship. | A |
| Mentors should educate mentees by various styles of mentoring in order to create learning environment. They can also select appropriate style based on available time, resources and mentees' preference. | A |
| The managers should realize that time constrains and limited mentors make it difficult to implement effective mentorship. The nursing home should define work accountability of mentor and mentee to allocate time reasonably. | A |
| Managers should make mentors and mentees aware of the benefits of the mentorship programs and motivate them to engage in the programs. | A |
| The management should support the mentorship programs strongly and establish rewarding mechanisms, which can enhance the engagement of mentors and mentees. | A |
| The management should realize that the old hierarchy can hinder mentorship programs. If mentors and mentees are in the same position, the trust in the relationship may be reduced. In such cases, managers should empower mentors to reduce the influence of the old hierarchy on the mentorship programs. | A |
